# ﻿A new species of *Begonia* (Begoniaceae) from the eastern mountains of Panama

**DOI:** 10.3897/phytokeys.256.145404

**Published:** 2025-05-13

**Authors:** Lucila Guillén, Peter W. Moonlight, Orlando A. Jara-Muñoz, María Sánchez de Stapf, Juan F. Carrión

**Affiliations:** 1 Herbario de la Universidad de Panamá, Facultad de Ciencias Naturales, Exactas y Tecnología, Campus Octavio Méndez Pereira, Panama, Panama Herbario de la Universidad de Panamá, Facultad de Ciencias Naturales, Exactas y Tecnología Panama Panama; 2 Botany, School of Natural Sciences, Trinity College Dublin, Dublin, Ireland Botany, School of Natural Sciences, Trinity College Dublin Dublin Ireland; 3 Royal Botanic Garden Edinburgh, 20A Inverleith Row, Edinburgh, EH3 5LR, UK Royal Botanic Garden Edinburgh Edinburgh United Kingdom; 4 Instituto de Ciencias Naturales, Universidad Nacional de Colombia, Carrera 30 No. 45-03, Building 425, Bogotá, D.C., Colombia Universidad Nacional de Colombia Bogotá Colombia; 5 Departamento de Botánica, Facultad de Ciencias Naturales, Exactas y Tecnología, Universidad de Panamá, Campus Octavio Méndez Pereira, Panama, Panama Universidad de Panamá Panama Panama; 6 Sistema Nacional de Investigación (SNI), SENACYT, Ciudad del Saber, Panama, Panama Sistema Nacional de Investigación (SNI) Panama Panama

**Keywords:** Biogeographic isolation, Cerro Chucantí, Darién, *Lepsia*, Serranía de Majé

## Abstract

*Begoniachucantiensis***sp. nov.** is described as a new species of BegoniasectionLepsia from Cerro Chucantí, Darién Province, in eastern Panama. A study of the literature and herbarium specimens, as well as fieldwork, suggests that the new taxon is distinguished from other species in the section by the combination of the following characters: uniform nodes, stipules persistent, 3.3–6 × 0.8–1.7 cm leaf blades, only two flowers per inflorescence, 12–14 stamens, and fruits with wings of similar shape and size. Images, information on geographic distribution, habitat, and a preliminary assessment of the conservation status of the new species are also provided.

## Abstract (es)

Se describe a *Begoniachucantiensis***sp. nov.** como una nueva especie de *Begonia* sección *Lepsia* del Cerro Chucantí, provincia de Darién, en el este de Panamá. Un estudio de la literatura y especímenes de herbario, así como trabajo de campo, sugiere que el nuevo taxón se distingue de las otras especies de la sección por la combinación de los siguientes caracteres: nudos uniformes, estípulas persistentes, láminas foliares de 3.3–6 × 0.8–1.7 cm, solo dos flores por inflorescencia,12–14 estambres y frutos con alas similares en forma y tamaño. También, se proporcionan imágenes, información sobre la distribución geográfica, el hábitat y una evaluación preliminar del estado de conservación de la nueva especie.

## ﻿Introduction

*Begonia* L. (Begoniaceae), with 2164 species (Hughes et al. 2015–), is the eighth most diverse plant genus (Moonlight et al. 2024). It exhibits a pantropical distribution and is easily distinguished by the following combination of characters: alternate, stipulate, and asymmetrical leaves; unisexual flowers, with inferior ovary that are usually three-winged (Moonlight et al. 2018).

Within *Begonia*, species are grouped into 74 sections, relatively few of which have been revised (Hughes et al. 2015–; Moonlight et al. 2018). One of these sections in the American Tropics is *Lepsia* (Klotzsch) A.DC., which comprises eight species characterized by an erect habit, absence of indumentum, pinnate, indistinct venation, four tepals in the male flower and five in the female flower, and axile placentation (Moonlight et al. 2018). Species in this section are primarily distributed in the Andes, from Venezuela to central Peru, with the greatest diversity in the northern Andes of Colombia. The only species recorded beyond the Andes to date is *Begoniaguaduensis* Kunth. This species has a peri-Amazonian distribution in Brazil, the Guiana Shield, and extends to Panama and Costa Rica (Moonlight et al. 2023).

According to the Catalog of Vascular Plants of Panama (Correa et al. 2004), *Begonia* comprises 36 species in the country. However, the current database of the University of Panama Herbarium (PMA) reports 42 species of this genus (unpublished data) and the *Begonia* Resource Centre reports 43 species in 11 sections (Hughes et al. 2015–). In all three cases, the section Lepsia is represented only by *B.guaduensis*.

The Cerro Chucantí Private Natural Reserve, established in 2006, occupies an area of approximately 750 ha, between the borders of the provinces of Panama and Darién (eastern Panama) (Camaño et al. 2019). Within this reserve is Cerro Chucantí, which, reaching 1439 m a.s.l., is the highest elevation of the Serranía de Majé. This area belongs to the Majé group, a geological formation of volcanic rocks dating back to the Tertiary Period and is considered among the oldest areas of the Isthmus of Panama (ANAM 2010).

Since the 1980s, the Chucantí region has been viewed as an area of high endemism (Ortiz et al. 2016). However, it remains a poorly explored area in Panama. Biodiversity inventories of the area, conducted in recent decades, have revealed taxonomic novelties in various taxonomic groups of animals, such as arthropods (Miranda and Bermúdez 2010; Bezark et al. 2013; Martins and Galileo 2013; Pinto-da-Rocha and Bragagnolo 2017; Vencl et al. 2017; González and Větrovec 2021; Longino and Branstetter 2021); amphibians (Batista et al. 2014, 2016a; Mebert et al. 2022) and reptiles (Batista et al. 2016b). Additionally, several angiosperm species from different families have been found, including Acanthaceae (Daniel and Vargas 2024), Araceae (Ortiz et al. 2016, Ortiz and Croat 2021; Croat et al. 2022), Heliconiaceae (Flores et al. 2017), Orchidaceae (Dressler 2003) and Rubiaceae (Flores et al. 2018).

During a floristic survey conducted in this area, a species of *Begonia* from section Lepsia was collected that did not resemble any other known taxon, so in this paper we describe it as a species new to science.

## ﻿Material and methods

Specimens of the new species were collected within the Cerro Chucantí Private Natural Reserve, characterized by a sub-equatorial climate with a pronounced dry season. The region experiences an average annual temperature ranging from 26.5 to 27.5 °C and decreases to 20 °C at higher elevations. Annual precipitation surpasses 2500 mm (ANAM 2010). Based on the Vegetation Map of Panama (ANAM 2000), the study area is classified as both submontane and tropical cloud forest. Collected specimens were processed and incorporated into the herbarium of the Universidad de Panamá (PMA). Floral and fruiting structures were preserved in 70% ethanol for subsequent measurements. Vegetative characteristics were quantified using an Olympus SZ 51 stereomicroscope based on herbarium material. Morphological comparisons were conducted with closely related species. The distribution map was generated using ArcGIS Pro.

## ﻿Taxonomic treatment

### ﻿ Begonia chucantiensis

Taxon classificationPlantaeCucurbitalesBegoniaceae

Guillén, Moonlight & Jara sp. nov.

ECAFEEC4-AF9B-519F-8E0B-8677E547AB0F

urn:lsid:ipni.org:names:77361668-1

Figs 1 , 2

#### Diagnosis.

Within BegoniasectionLepsia, *B.chucantiensis* resembles *B.confinis* in having persistent stipules and two flowers per inflorescence; however, it differs by having glabrous young branches and uniform nodes (vs. ferruginous indumentum and swollen nodes), stipules 8.5–10.5 × 3.5–4.5 mm (vs. 0.8–1.3 × 0.3–0.4 mm), elliptic to lanceolate leaf blades (vs. rhomboid subtrilobed), 3.3–6 × 0.8–1.7 cm leaf blades (vs. 0.6–1.3 × 0.3–0.6 cm), peduncles up to 1.3 cm (vs. 2.8 cm), 12–14 stamens (vs. 8–10), and fruits with similar wing shape and size (vs. one wing conspicuously unequal).

#### Type.

**Panama** • **Darién**: Distrito de Santa Fé. Reserva Natural Privada Cerro Chucantí; 8°47'54"N, 78°27'46"W, 1297 m a.s.l., 27 Mar 2023 (♂ fl., ♀ fl., and fr.), *Mitre-Ramos, C., Camaño, J., Ortega, J. & Ortega, B.* 8 (holotype: PMA132927! [isotypes will be sent to the following herbaria: COL, K, MO]).

**Figure 1.**
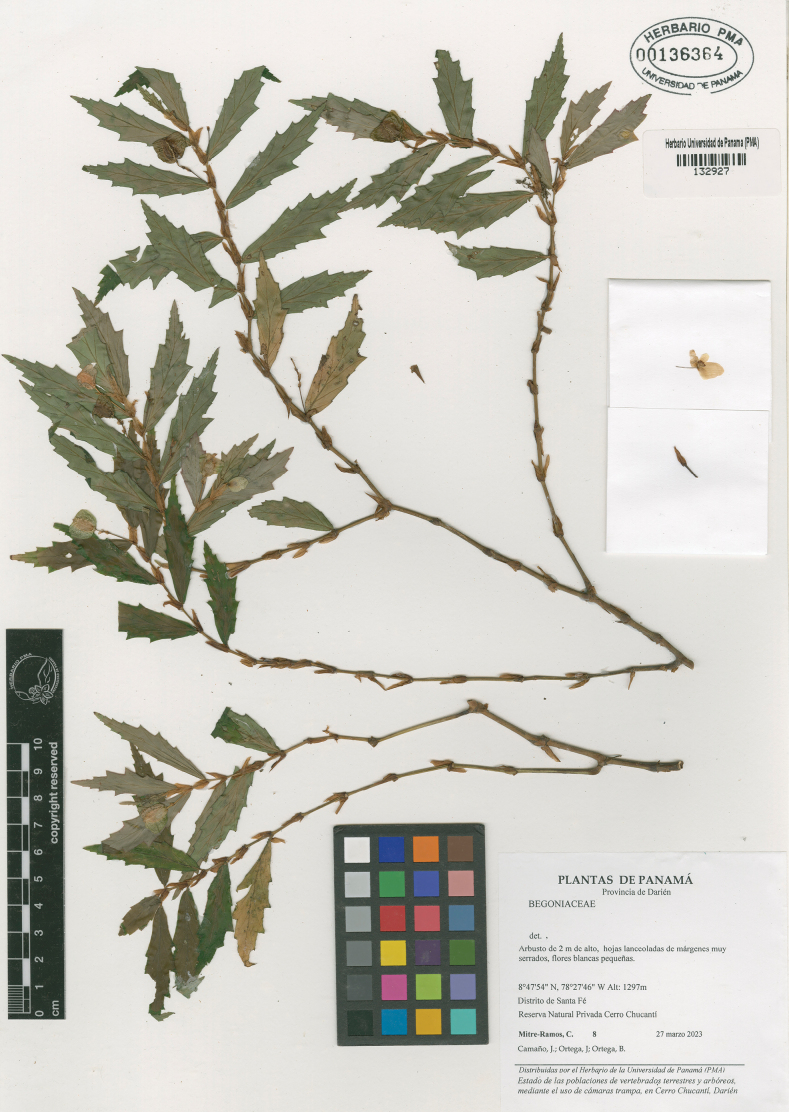
Holotype of *Begoniachucantiensis* sp. nov. (PMA132927).

#### Description.

Perennial, terrestrial, erect herb up to 2 m tall. Main stem green, succulent, glabrous, branched; branches slender, lenticels absent, internodes glabrous, 0.5–4 cm long and 1.2–3.5 mm in diameter, nodes not swollen. Stipules persistent, pink to brown, oblong to ovate, 8.5–10.5 × 3.5–4.5 mm, slightly asymmetric, glabrous, membranous, hyaline, margin entire, apex obtuse, with a central vein and an apical seta. Leaves more than 5 per stem; petioles green, 0.5–1.5 mm × 0.3–0.7 mm, terete, glabrous; leaf blade elliptic to lanceolate, 3.3–6 × 0.8–1.7 cm, discolorous, lustrous green adaxially, paler green abaxially, with slightly pinkish veins and margin, base slightly asymmetric, apex acuminate, margin dentate, each dent with a hydathode and often an additional hydathode between the dents, dent triangular, 0.6 × 0.3 mm; venation pinnate, 5–8 veins per side. Inflorescences bisexual, axillary, cymose, 2-flowered, protandrous, subtended by a persistent, dissected bract, 7–8 × 6–7 mm, margin serrate; peduncle up to 1.3 cm long. Staminate flowers: pedicel up to 1.2 cm long; tepals 4, white, glabrous, margin entire, apex rounded, outer 2 ovate, 10–12 × 7–9 mm, apex rounded, inner 2 oblanceolate, 7–9 × 4–5 mm; stamens 12–14, yellow, filaments 0.5–1 mm long, free, anthers ellipsoid, 2.7–3.3 × 0.8–1 mm, dehiscence by lateral slits, connective 0.9–1 × 0.7–0.9 mm, basifixed. Pistillate flowers: pedicel up to 4 mm long, bracteoles 2, persistent, positioned directly below the ovary, ovate, 5–7 × 3.5–4.5 mm, auriculate, margin dentate; tepals 5 or 6, unequal, deciduous in fruit, ovate, 9–14 × 4–9 mm, white, glabrous, margin entire; ovary body ovoid, 6–8 × 3–4 mm, whitish, translucent, glabrous, 3-winged, wings nearly equal in size, 6–8 × 2.5–3.5 mm, margin serrate; 3-locular, placentas bifid and bearing ovules on both surfaces; styles 3, yellow, united at base in a 0.8–1.2 mm long column; arms 1–2 mm long, once divided; stigmatic papillae in a spirally twisted band. Fruits: pedicels up to 2 cm long; body ovoid, up to 10 × 4 mm, drying brown, wings expanding up to 12 × 7 mm. Seeds 0.3–0.5 × 0.1–0.2 mm, ellipsoid.

**Figure 2.**
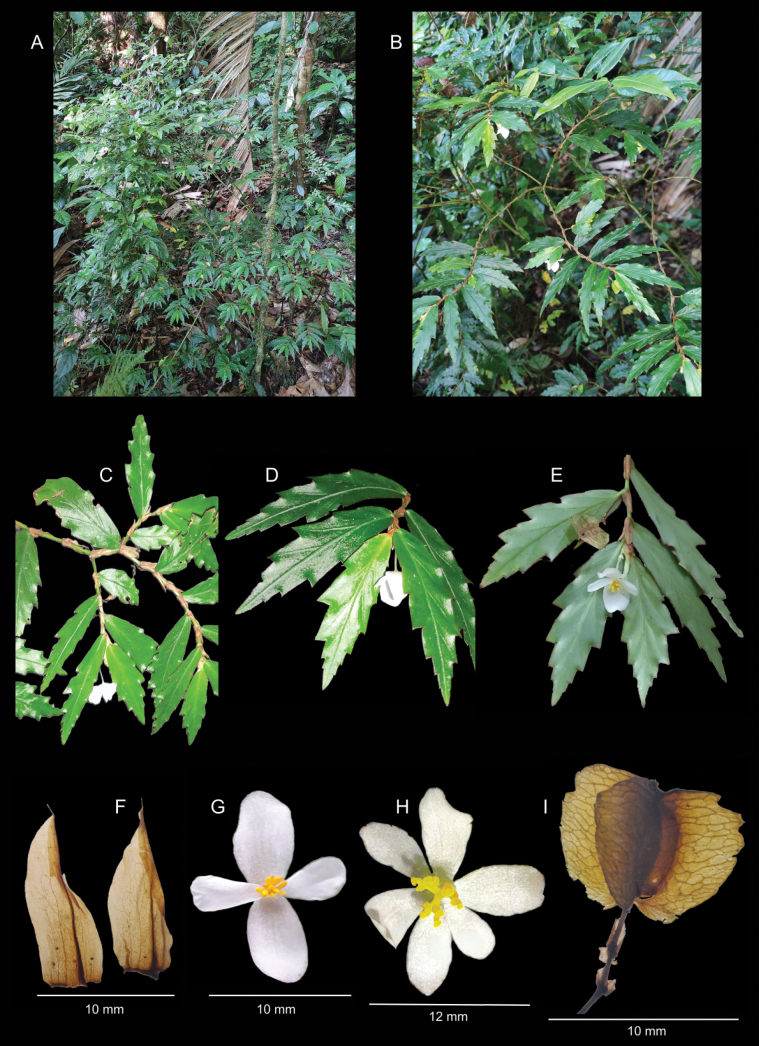
Morphological aspects of *Begoniachucantiensis* sp. nov. **A** plant habit **B** fertile branches **C** detail of fertile branch **D** leaves viewed from the adaxial side and staminate flower **E** leaves viewed from the abaxial side, staminate flower and fruit **F** stipules **G** front view of staminate flower **H** pistillate flower **I** lateral view of the fruit. Images **A, B, D, E** C. Mitre-Ramos. **C, F, I** L. Guillén. **G, H** J.L. Guerra.

#### Distribution.

**Figure 3.**
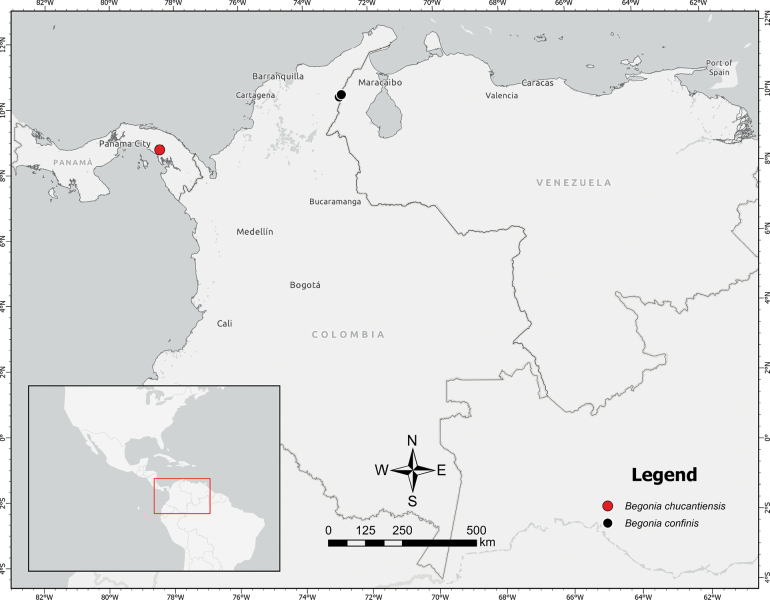
Geographical distribution of *Begoniachucantiensis* sp. nov. (red circle) in relation to its most morphologically similar relative, *B.confinis* (black circles).

*Begoniachucantiensis* is only known from the type locality, Cerro Chucantí (Serranía de Majé), in eastern Panama (Fig. 3).

#### Habitat and ecology.

The species grows in the understory, on steep slopes, beneath 20–30 m tall trees, in premontane rainforest, at an elevation of 1200–1300 m above sea level. It has been recorded with flowers and fruits in January and March.

#### Etymology.

The specific epithet refers to the locality where the new species is known: Cerro Chucantí.

#### Preliminary conservation status.

*Begoniachucantiensis* is currently known from only two localities that we consider to be one location (Area of Occupancy [AOO] of approximately 4 km^2^) within the Cerro Chucantí Private Natural Reserve. Although confined to a private reserve, *B.chucantiensis* faces significant threats. Long-term protection is not assured, and any adverse event, including extreme weather, could lead to the loss of the entire population. Furthermore, deforestation and agricultural expansion pose substantial risks in the surrounding areas, resulting in a projected decline in both habitat quality and AOO (<10 Km^2^). Consequently, following the IUCN Red List Categories and Criteria (IUCN 2024), this species is preliminarily classified as Critically Endangered (CR B2ab(iii)).

#### Additional specimen examined

**(paratype). Panama** • **Darién**: Distrito de Santa Fé. Serranía de Majé. Corregimiento de Río Congo. Reserva Natural Privada Cerro Chucantí, laderas de Cerro Chucantí, cercano al campamento del filo, 8°48'16"N, 78°27'34"W, 1300 m a.s.l., 27 Jan 2023 (♂ fl., ♀ fl., and fr.), *Guillén, L., Guerra, J.L., Lino, M. & Fatacioli, G. 783* (PMA!).

#### Discussion.

Following the taxonomic key for *Begonia* in the Flora Mesoamericana (Burt-Utley 2015), the new species would be placed in couplet 23, which separates *B.liesneri* Burt-Utley & Utley and *B.semiovata* Liebm. Both species share some characters with *B.chucantiensis*, including: the habit of an erect to somewhat sprawling subshrub; apparently non-rooting stems at the nodes; persistent to lately deciduous stipules; glabrous to very sparsely pilose or villous and pinnately veined glabrous blades; well-developed, persistent bracteoles and sub-equal ovary/fruit wings. The new species shares with *B.liesneri* the subshrubby habit and staminate flowers with tepals greater than 7 mm long. *B.semiovata*, on the other hand, is an herbaceous species with staminate flowers with tepals up to 3 mm long. On the other hand, *B.semiovata* presents leaves with a similar number of veins per side (7–9) as *B.chucantiensis* (5–8), while *B.liesneri* has more veins per side ((10)12–15).

##### ﻿Supplement to the key to *Begonia* in Flora Mesoamericana (Burt-Utley 2015)

Note that couplet 23 has been modified, and a new couplet, 23’, has been inserted, with characters to differentiate *B.liesneri* from *B.chucantiensis*.

**Table:** 

22	Deciduous, rudimentary bracteoles; unequal ovary and capsule wings	***B.seemanniana***
–	Persistent, well-developed bracteoles; subequal ovary and capsule wings	**23**
23	Annual herb, up to 0.3 m tall; staminate tepals 2–3 mm long; fruiting pedicel to 7 mm long	***B.semiovata***
–	Perennial herb, up to 2 m tall; staminate tepals 7–16 mm long; fruiting pedicel to 24 mm long	**23**’
23'	Leaf blade 7–13 × 1.8–4.3 cm; inflorescences 7–10-flowered; staminate flowers with ca. 23 stamens	***B.liesneri***
–	Leaf blade 3.3–6 × 0.8–1.7 cm; inflorescences 2-flowered; staminate flowers with 12–14 stamens	***B.chucantiensis*** ^1^

In BegoniasectionLepsia, where we place the new species, 8 species are currently recognized: *B.barrigae* L.B.Sm. & B.G.Schub., *B.confinis* L.B.Sm. & Wassh., *B.foliosa* Kunth, *B.guaduensis* Kunth, *B.holtonis* A.DC., *B.meridensis* A.DC., *B.opuliflora* Putz and *B.praerupta* Irmsch (Moonlight et al. 2023). However, *B.chucantiensis* is readily distinguished from most other members of the Lepsia section by its 2-flowered inflorescences. While most species within the section possess multifloral inflorescences with more than 12 flowers, only *B.confinis* shares this bifloral characteristic. Nevertheless, *B.chucantiensis* can be differentiated from *B.confinis* by the characteristics mentioned in the diagnosis. Furthermore, *B.confinis* is geographically restricted to the Serranía del Perijá, the northernmost extension of the Andes mountain range along the Colombia-Venezuela border (Fig. 3), while *B.chucantiensis* is found over 600 km away on Cerro Chucantí in the Serranía de Majé, eastern Panama.

The new species shares some similarities with the Brazilian *Begoniaitatinensis* Irmsch. ex Brade (Begoniasect.Pritzelia (Klotzsch) A.DC.), including persistent stipules, small, slightly asymmetric leaves with pinnate venation, 2-flowered inflorescences, and 8–10 stamens. However, species in section Pritzelia have cystoliths in the leaf epidermal cells (Moonlight et al. 2018), which are absent in this new species. Additionally, *B.itatinensis* has a scandent, pubescent stem (Smith and Smith 1971; Jaramillo 2017) and entire placentas (Brade 1944; Mamede et al. 2012), while *B.chucantiensis* has an erect, glabrous stem and bifid placentas.

## Supplementary Material

XML Treatment for Begonia chucantiensis

## Footnotes

The Flora Mesoamerica key continues at couplet 24.

## ﻿Additional information

### Author ORCIDs

Lucila Guillén https//orcid.org/0009-0007-2416-8130

Peter W. Moonlight https//orcid.org/0000-0003-4342-2089

Orlando A. Jara-Muñoz https//orcid.org/0000-0002-7123-124X

María Sánchez de Stapf https//orcid.org/0000-0002-8095-5899

Juan F. Carrión https//orcid.org/0000-0003-4015-375X

### Data availability

All of the data that support the findings of this study are available in the main text
